# Isolation of circulating endothelial cells provides tool to determine endothelial cell senescence in blood samples

**DOI:** 10.1038/s41598-024-54455-5

**Published:** 2024-02-21

**Authors:** Katrin Kalies, Kai Knöpp, Leonie Wurmbrand, Laura Korte, Jochen Dutzmann, Claudia Pilowski, Susanne Koch, Daniel Sedding

**Affiliations:** 1 Mid-German Heart Center, Department of Internal Medicine III, Division of Cardiology, Angiology and Intensive Medical Care, University Hospital Halle, Martin-Luther-University Halle-Wittenberg, Ernst-Grube-Strasse 40, 06120 Halle (Saale), Germany; 2 Department of Cardiology and Angiology, Hannover Medical School, Carl-Neuberg Straße 1, 30625 Hannover, Germany

**Keywords:** Cardiovascular biology, Senescence

## Abstract

Circulating endothelial cells (CEC) are arising as biomarkers for vascular diseases. However, whether they can be utilized as markers of endothelial cell (EC) senescence in vivo remains unknown. Here, we present a protocol to isolate circulating endothelial cells for a characterization of their senescent signature. Further, we characterize different models of EC senescence induction in vitro and show similar patterns of senescence being upregulated in CECs of aged patients as compared to young volunteers. Replication-(ageing), etoposide-(DNA damage) and angiotensin II-(ROS) induced senescence models showed the expected cell morphology and proliferation-reduction effects. Expression of senescence-associated secretory phenotype markers was specifically upregulated in replication-induced EC senescence. All models showed reduced telomere lengths and induction of the INK4a/ARF locus. Additional p14ARF-p21 pathway activation was observed in replication- and etoposide-induced EC senescence. Next, we established a combined magnetic activated- and fluorescence activated cell sorting (MACS-FACS) based protocol for CEC isolation. Interestingly, CECs isolated from aged volunteers showed similar senescence marker patterns as replication- and etoposide-induced senescence models. Here, we provide first proof of senescence in human blood derived circulating endothelial cells. These results hint towards an exciting future of using CECs as mirror cells for in vivo endothelial cell senescence, of particular interest in the context of endothelial dysfunction and cardiovascular diseases.

## Introduction

Endothelial cell senescence has been shown to contribute to endothelial dysfunction by accelerating the impairment of cellular function, barrier disruption and extracellular matrix degradation^1^. Senescence in general and in endothelial cells (EC) can be induced by multiple (patho-)physiological mechanism, including aging and age-related disorders^2^, chronic cellular stress (oxidative and mechanical)^3–5^, DNA damage (e.g. in cancer) or telomere shortening due to altered telomerase function (also frequently observed in cancer)^6,7^. Further, senescence-inducing factors might be therapy related as to chemotherapy or due to risk factors as smoking^8,9^. As such, studying EC senescence and its inducing mechanisms are of great interest to various research fields. However, the complex nature of senescent EC physiology, the magnitude of different cellular pathways involved and difficulties with accessing material for functional studies, complicates endothelial cell senescence research^10,11^.

In this regard, circulating endothelial cells (CEC) present a promising tool to further unravel EC characteristics. Currently exploited as biomarkers of EC function and cardiovascular disease (CVD) progression, CECs are emerging as easily accessible reflections of in vivo EC function^12,13^. CEC are defined as non-haematological cells with a mature endothelial identity and a limited growth capacity. They detach from the endothelium of the blood vessel wall after differentiation or vascular damage^12,14^. Thereby, CEC differ from endothelial progenitor cells (EPC) which are derived from the bone marrow to differentiate into mature endothelial cells and have a major role in vascular repair^15^. So far, the direct cell count of CEC has been exploited as a hint to the amount of endothelial damage^16^. However, the direct count displays a wide range in different studies due to different isolation protocols. Further, low CEC yield in human blood hampers CEC senescence research^16^. Previously published papers only agreed on the common expression of CD146 by CEC, sometimes accompanied by others among them CD45−, CD105+, vWF+, CD31+, CD133−, and CD34+^17,18^. Extending on this trend, we suggest CEC’s to be usable as biomarkers of endothelial cell senescence and vascular ageing and thereby to endothelial dysfunction.

First, we here present a detailed characterization of endothelial cells in three different senescence models in vitro, namely replicative senescence, DNA-damage induced senescence and oxidative stress regarding their molecular features. In a next step we established a novel method of CEC isolation based on a combination of magnetic bead depletion (MACS) and fluorescence activated cell sorting (FACS) analysis to enumerate the cells enabling to provide first proof of CEC senescence in aged as compared to young volunteers.

## Methods

### Human material

Human EDTA-blood was collected from young volunteers and aged patients with CVD. Informed consent was obtained from all participants. The study was approved by the guidelines of the ethics committee from the Medical Faculty of the Martin-Luther-University Halle-Wittenberg (Registration number 2019-144) and performed according to the guidelines of the ethics committee from the Medical Faculty of the Martin-Luther-University Halle-Wittenberg and the Declaration of Helsinki. Isolated material was stored for further use. The probands were recruited by the University Hospital Halle, Department for Internal Medicine III. The healthy young participants were required to have a normal echocardiogram to meet the inclusion criteria, while the aged cohort (> 60 years) needed either an abnormal echocardiogram or confirmation of CVD through a (prior) cardiac catheterization. Both groups were excluded if they had experienced an acute myocardial infarction or significant cardiovascular event within the past 3 months, were within 30 days after cardiopulmonary resuscitation, or if they had acute or chronic infectious diseases, along with concurrent autoimmune or tumor diseases. None of the probands underwent surgical interventions prior to blood collection. In total, 8 ml blood was collected in the morning and processed directly for further analysis.

### Cell culture and models of senescence

Human umbilical vein endothelial cells (HUVEC) were purchased from Lonza and cultivated in endothelial cell growth medium (PromoCell). All cells were maintained at 37 °C and 5% CO_2_ in a humidified incubator, grown to confluency and passaged in a 1:3 ratio. For replication-induced senescence, passages < 5 and > 15 passages (Passage doubling level (PDL) < 6 respectively > 42) were considered. For treatment-induced senescence, cells of early passage numbers (< 5) were treated either with 150 µM Angiotensin II (AngII, 48 h) or with 100 µM Etoposide (12 h).

### Isolation of RNA and quantitative real-time PCR (qRT-PCR)

Total RNA-isolation of cell culture samples was performed using the RNeasy Mini Kit together with the QIAshredder columns (Qiagen) following manufacturer’s instruction. Cells were scraped in RLT-buffer, centrifuged over a shredder column and then transferred to an RNA- binding column. After several washing steps, RNA was eluted in Nuclease-free water. RNA measurement was performed using a nanodrop. RNA was transcribed to cDNA using the High Capacity cDNA Reverse Transcription kit (Applied Biosystems). For the qRT-PCR the Blue S’Green qPCR Kit from Biozym was used according to manufacturer’s instruction. A list of used primers is shown in supplementary Table S1.

Total RNA-isolation of circulating endothelial cells from blood samples was performed using the miRNeasy micro Kit (Qiagen) following manufacturer’s instructions for low cell counts. Briefly, cells were resuspended in Trizol, homogenized by vortexing, incubated for 5 min and then vigorously mixed with chloroform. After a short incubation step, samples were centrifuged for 15 min at 12,000×*g* at 4 °C to achieve a phase separation. The upper aqueous phase was transferred and thoroughly mixed with 1.5 volumes of 100% ethanol. The sample was then applied to an RNA-binding column. After several washing steps where the buffer was prepared with isopropanol instead of ethanol as well as an DNA digestion step, the spin column was washed with 80% ethanol. Followed by a short centrifugation step to dry the membrane, the RNA was eluted in Nuclease-free water.

### Determination of proliferation and telomere length

Proliferation was assessed by a Bromodeoxyuridine (BrdU) assay (Cell proliferation ELISA (colorimetric) (Roche)) according to the manufacturer’s instructions. Further, live cell count was determined by life cell imaging. Therefore, cells were seeded into 96-well flat bottom plates. After cell adhesion, the plate was inserted into the life cell imaging system Cytation 1. Images were taken every 30 min for a 24-h period. Number of cells in the brightfield image were identified and counted automatically by the Gen5 software.

Genomic DNA was isolated from the cells by following the manufacturer’s instructions of the GeneJET Genomic DNA Purification Kit (Thermo Fisher Scientific). Briefly, cells were diluted in lysis buffer with Proteinase K and incubated for 10 min at 56 °C. To ensure RNA digestion, RNase A was added for a prolonged cultivation time of 10 min at room temperature. Followed by the addition of 50% ethanol, the solution was transferred to a DNA purification column and centrifuged at 6000×*g* for 1 min, After several washing steps, the DNA was eluted. Further telomere length determination was performed using a Relative Human Telomere Length Quantification qPCR assay following manufacturer’s instructions (ScienCell Research Laboratories).

### Senescence-associated β-galactosidase (SA-β-gal) staining

For the senescence-associated β-galactosidase staining we used a protocol previously published by Debacq^19^. For the colorimetric assay, cells were seeded on chamberslides to 50% confluency, fixed for 10 min and incubated over-night with an X-Gal staining solution.

### Immunofluorescence staining and morphometric analysis

For immunofluorescence staining’s, cells were seeded to 80% confluency in 8-well microscopy slides. 24 h after seeding, cells were fixed for 30 min and blocked for 1 h. Staining with the primary antibody was performed overnight at 4 °C, the secondary antibody staining was conducted at room temperature for one hour. Cells were stained with DAPI and prepared for microscopy. Microscopic images were analyzed using ImageJ to evaluate cell size and nuclear size. Further information for the used antibodies can be found in Table S2.

### CEC isolation from human blood samples

First, the collected human EDTA-blood was subjected 1:20 to red blood cell lysis buffer (Biolegend) for 15 min. After centrifugation (345*g for 5 min), the cell pellet was resuspended in phosphate buffered saline (PBS) with 1% bovine serum albumin (BSA) and 1 mM Ethylenediaminetetraacetic acid (EDTA). In the following step, cells were incubated with CD45-magnetic beads for 15 min at 4 °C (Miltenyi Biotec), washed, and CD45 negative cells were depleted with LD-columns in the magnetic field of a MidiMACS-Seperator (Miltenyi). Followed by an additional washing step, the effluent was subsequently stained with fluorescent labeled antibodies for CD45, CD11b, CD31, CD146 and CD34 (see supplementary Table S3). After 15 min of incubation at 4 °C in the dark, the sample was centrifuged, resuspended and sorted with a cell sorting system in the core facility of the University hospital Halle. CD45dim/CD11b−/CD31+/CD146+/CD34+ cells were defined as circulating endothelial cells. For further information on RNA and DNA isolation see above. For these analysis, cells were directly sorted into the corresponding lysis buffer. qRT-PCR analysis was performed as described above for mRNA expression analysis of endothelial cell and senescence markers as well as for telomere length determination. Additionally, freshly isolated endothelial cells were seeded on Fibronectin (2 µg/cm^2^) and stained for endothelial cell markers, to confirm the gating strategy. Immunofluorescence staining was performed as described above.

### Statistical analysis

Statistical analysis of each experiment was performed with GraphpadPrism 8. All shown data are presented as mean ± standard deviation, n indicates the number of individual experiments. Presented in vitro data are further color coded to the respective condition: replicative senescence in grey, Etoposide treatment in red and Angiotensin II treatment in blue. All datasets were normalized to their respective control. P < 0.05 was considered statistically significant. Differences between the study groups were analyzed by two-tailed unpaired Student’s t-test.

## Results

### Senescent ECs present with reduced telomere lengths and heterogeneous SASP induction independent of EC senescence model used

The distinct role of senescent endothelial cells and their contribution to the development of endothelial dysfunction which in turn is involved in most cardiovascular pathologies is still under investigation. Oxidative stress, DNA damage and telomere stress (e.g. ageing) have been identified as main drivers of EC senescence, activating distinct cell cycle arrest mechanisms. As such, a multitude of methods to define senescent cells are currently described in literature. In order to establish relevant methods for EC senescence, we first evaluated standardly used methods of senescence detection for three distinct models of EC senescence: replication-induced, etoposide-induced and angiotensin II-induced EC senescence representing ageing, DNA damage and oxidative stress-induced senescence respectively.

**Figure 1:**
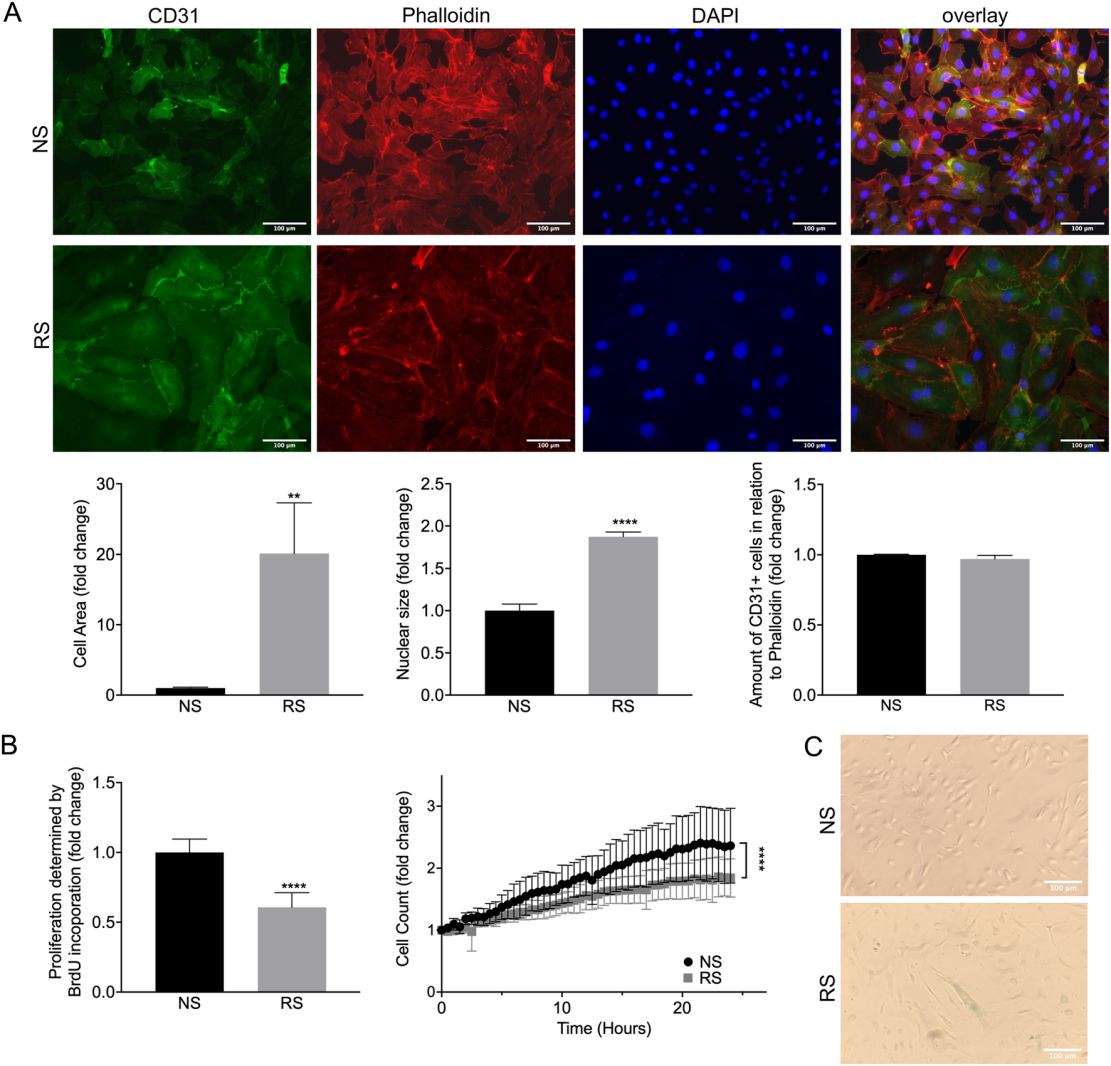
Replicative senescence (RS) on morphometric and proliferation scale. (**A**) Immunofluorescence staining of non-senescent (NS) and replicative senescent (RS) cells with staining for CD31 (green), Phalloidin (red) and DAPI (blue). Determination of cell area by measuring Phalloidin area in comparison to cell count. n = 3 **P = 0.0018. Determination of nuclear size by measuring DAPI stained area. n = 3 ****P < 0.0001. Amount of CD31^+^ cells in relation to Phalloidin stained cells n = 3 P = 0.1205. (**B**) Proliferation determined by BrdU incorporation. n = 3 ****P < 0.0001. Determination of proliferation by life cell imaging and cell counting over 24 h. n = 3 ****P < 0.0054. (**C**) Senescence-associated β-galactosidase staining. Replicative senescent cells are stained light blue.

In our hands, all models presented with the expected increased cell size and nuclear size, a flattened appearance as well as reduced proliferation as established by BrdU incorporation and cell count over time (Fig. 1A and B, data not shown). The detectable co-staining with the endothelial marker CD31 illustrates that also at higher passages, cells remain endothelial cells (Fig. 1). As described earlier^19,20^, beta-galactosidase positive cells accumulated in HUVECs at high passage numbers in the replication-induced EC senescence model (Fig. 1C).

Having established senescence induction on morphometric and proliferation scale, we further focused onto characterizing the distinct senescent pathways induced in these models.

**Figure 2:**
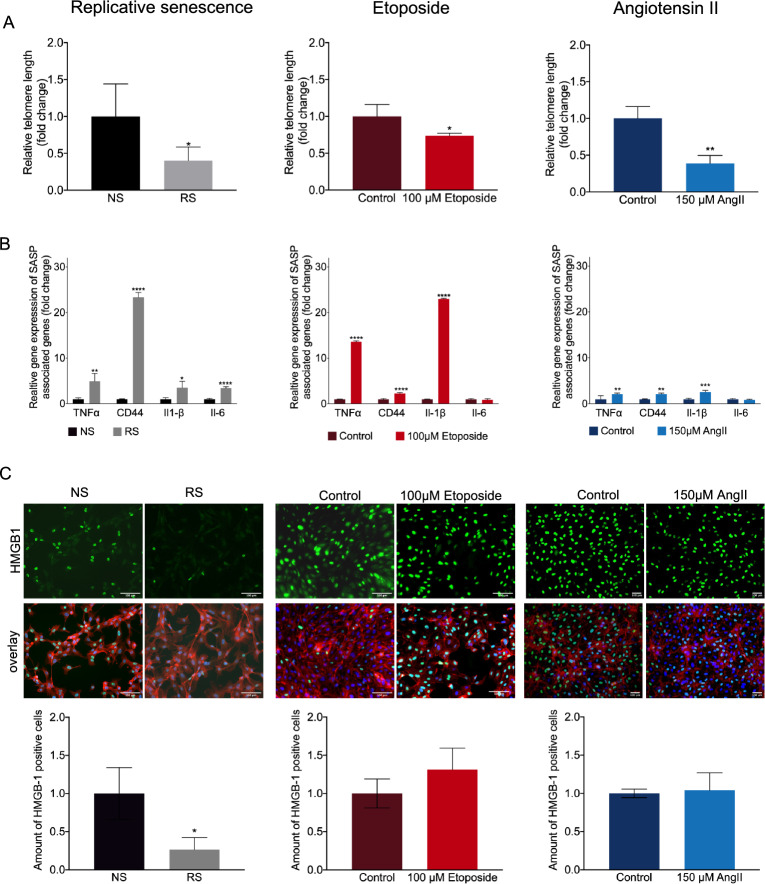
Measurement of telomere length, genes associated with the senescence-associated secretory phenotype and visualization of HMGB-1. (**A**) Quantification of relative telomere length by qRT-PCR analysis in replicative senescent (RS) cells. n = 3 *P = 0.0201. Relative telomere length in the Etoposide-treated cells. n = 3 *P = 0.0499. Relative telomere length in the angII-induced senescent cells. n = 3 **P = 0.0054. (**B**) Relative gene expression level of CD44, TNFα, IL-1β and IL-6. Replicative senescent cells (RS) n = 3 CD44 ****P < 0.0001, TNFα **P = 0.0043, IL-1β *P = 0.0119 and IL-6 ****P < 0.0001. Etoposide-induced senescent cells. n = 3 CD44 ****P < 0.0001, TNFα ****P < 0.0001, IL-1β ****P < 0.0001 and IL-6 P = 0.8559. AngII-induced senescent cells. n = 3 CD44 **P = 0.0025, TNFα **P = 0.0025, IL-1β ***P = 0.0001 and IL-6 P = 0.9855 (C) Immunofluorescence staining for HMGB-1 (green), Phalloidin (red) and DAPI (blue). Replicative senescent cells (RS). n = 3 *P = 0.0274. Etoposide-induced senescent cells. n = 3 P = 0.1855 AngII-induced senescent cells. n = 3 P = 0.7804.

The length of the telomere is an important criterion when studying cellular age. Also in our hands, telomere length decreased in replication-induced senescent endothelial cells and Ang II-treated cells and to a lesser extent in etoposide-treated HUVECs (Fig. 2A).

Next to arrested cell growth, the senescence-associated secretory phenotype (SASP) is a key characteristic of cellular senescence, promoting age-associated inflammation and pathology. This SASP is however heterogeneous, with the exact composition depending on senescence-inducer present. Also in our hands, we observe enhanced, yet heterogenous, expression of SASP-associated genes tumor necrosis factor-αlpha (TNFα), Interleukin-1 beta (IL-1β) and CD44^21^ in all three EC senescence models tested, with etoposide treatment inducing most pronounced expression regulating effects. In turn, Interleukin-6 (IL-6) was specifically upregulated in replication-induced EC senescence only (Fig. 2B). In line with that, we observed decreased cellular presence of High mobility group box 1 (HMGB-1) as assessed by immunofluorescent staining in replication-induced senescent ECs (Fig. 2C). Upon senescence or cellular stress, HMGB-1 is shuffled into the extracellular space to where it binds amongst others cell surface to receptor for advanced glycation endproducts (RAGE) and toll-like receptors (TLRs) to initiate signaling, that results in expression of inflammatory cytokines including IL-6^22,23^. While we did not assess extracellular HMGB-1, we suggest that decreased cellular HMGB-1 observed here is likely reflecting increased shuffling of HMGB-1 into extracellular space, rather than reduced expression itself. For the other models a decreased presence of HMGB-1 was not observable.

### Replication- and etoposide-induced senescent ECs activate the p16INK4a cell cycle arrest pathway, while Ang II treatment likely acts via p14ARF-mediated senescence induction

**Figure 3:**
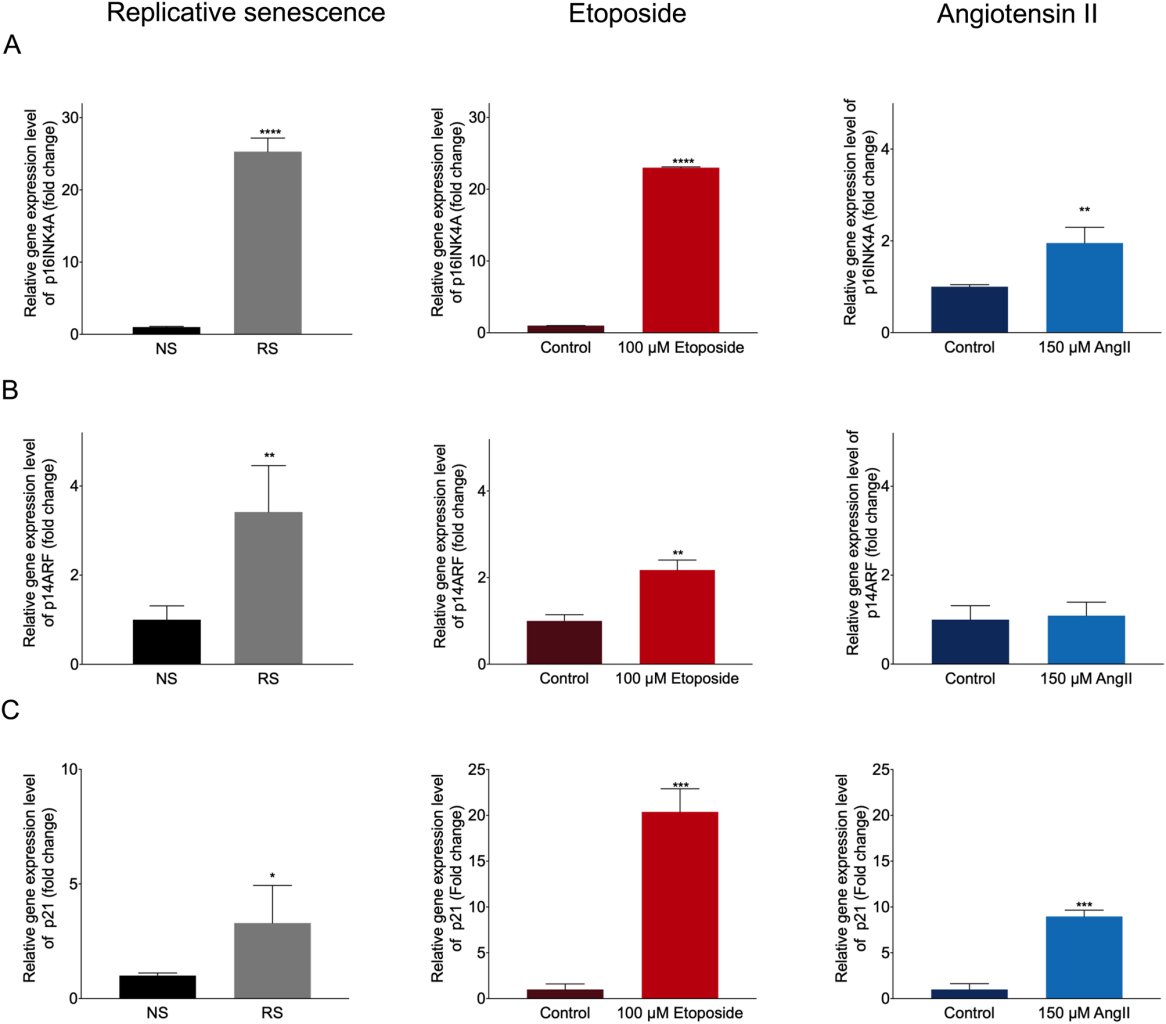
Quantification of cell cycle regulators p16INKA, p14ARF and p21 associated with cellular senescence. (**A**) Relative gene expression level of p16INK4A in all three models. Replicative senescent cells (RS) n = 3 ****P < 0.0001. Etoposide-induced senescent cells. n = 3 ****P < 0.0001. AngII-induced senescent cells. n = 3 **P = 0.0088. (**B**) Relative gene expression level of p14ARF in all three models. Replicative senescent cells (RS). n = 3 **P = 0.0044. Etoposide-induced senescent cells. n = 3 **P = 0.0017. AngII-induced senescent cells. n = 3 P = 0.7376. (**C**) Relative gene expression level of p21 in all three models. Replicative senescent cells (RS). n = 3 *P = 0.0317. Etoposide-induced senescent cells. n = 3 ***P = 0.0002. AngII-induced senescent cells. n = 3 ***P < 0.0001.

Growth arrest was observed in all three models of EC induction, as illustrated earlier. Here, we assessed key cell cycle regulators involved in senescence induction in our three models, namely the INK4a/ARF locus and downstream p21 activation. Indeed, p16INK4a expression was elevated in replication-induced senescent ECs, a trend that could be confirmed on mRNA level for etoposide- and to a lesser extent for AngII treated cells (Fig. 3A). P14ARF induction was observed for replication-induced and etoposide-treated cells, but not for AngII -mediated senescence induction (Fig. 3B), indicating that this mainly oxidative stress-based model does not activate the p14ARF-p53-p21 cell arrest pathway. Yet, p21 was highly expressed in all three models, thus also AngII-treated cells (Fig. 3C), suggesting that p21 induction in AngII-treated cells is potentially independent of p14ARF induction.

**Figure 4:**
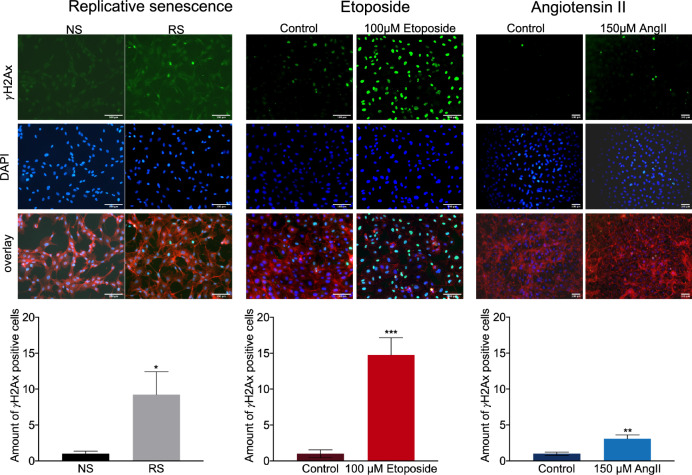
Impairment of cellular senescence on DNA damage. Immunofluorescent staining and quantification of γH2Ax (green) positive cells, Phalloidin (red), DAPI (blue). Replicative senescent cells (RS). n = 3 ****P < 0.0001. Etoposide-induced senescent cells. n = 3 ***P = 0.0006. AngII-induced senescent cells. n = 3 **P = 0.003.

Hinting towards a potential explanation, we observed increased DNA damage quantified as amount of histone H2A.X phosphorylation (γH2A.X) present in cells, in replication-induced senescent ECs, complimentary to expected DNA damage in etoposide-treated cells (Fig. 4), suggesting that p14ARF pathway induction is potentially mainly observed in EC senescence models with excessive DNA-damage.

### CECs from elderly patients show increased levels of senescence marker

**Figure 5:**
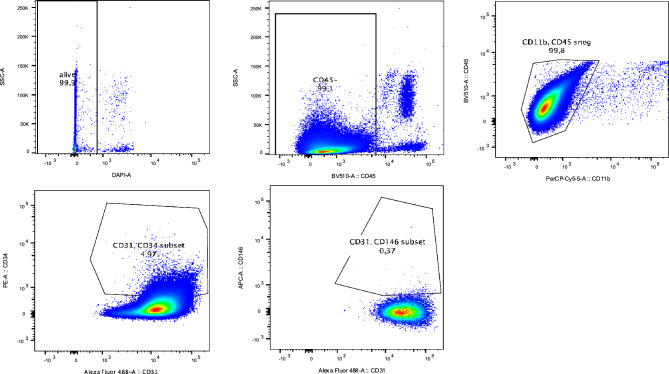
Isolation of circulating endothelial cells by combined selection with MACS-FACS-technology. Gating strategy for the FACS-analysis of circulating endothelial cells from human blood samples. Living cells were gated based on DAPI staining, followed by exclusion of CD45+ cells. After that, a gate for CD11b/CD45 was included. With this cell fraction the further discrimination was performed by CD31/CD34 gating and CD31/CD146 gating.

Next, we aimed to determine whether endothelial cell senescence can be detected in circulating endothelial cells, potentially reflecting in vivo endothelial dysfunction and vascular ageing. To this end, we first established a combined MACS-FACS-based CEC isolation protocol by integrating MACS-based CD45− cell selection combined with published and validated gating strategies of FACS^13,24–26^ (live cell selection followed by CD45−, CD11b−, CD31+, CD34+ and CD146+ selection). In a first step, cells were labeled with CD45−magnetic beads and the CD45− cells were depleted by magnetic sorting. Subsequently CD45 negative cells resulting from MACS pre-selection, were FACS-sorted. Briefly, living cells determined by DAPI negative cells were used for a CD45 negative (incl. CD45^dim^) selection, followed by a CD11b negative population. With this cell fraction the further discrimination was performed by CD31/CD34 gating and CD31/CD146 gating (Fig. 5).

As validation of our protocol, these cells were plated on fibronectin-coated plates and stained for endothelial cell markers CD31, Von Willebrand factor (vWF) and CD146 (Supplemental Fig. 1). Attempts to cultivate the circulating endothelial cells from aged patients on fibronectin failed as they did not attach to the fibronectin coated plate whereas the cells from the young controls did attach. This observation already gives a hint on different rest functionality and might indicate differences in gene expression.

In total, CECs were isolated from 11 young volunteers and 11 aged patients with CVD comorbidities (Table 1). The young volunteers had an average age of 25.2 years, whereas the aged cohort was 73.5 years aged. All aged patients presented with at least presence of one CVD risk factor as smoking, diabetes, hypertension or hyperlipidemia. From the aged cohort, 10 participants were diagnosed for coronary artery disease (CAD) and all the participants in this cohort were treated for CVD, mostly with ß-blockers, antiplatelet drugs and statine. In the young healthy cohort, all of the probands had a normal echocardiogram, however one proband was treated with Sodium/glucose cotransporter-2 (SGLT-2) inhibitors. Detected number of CEC had a low percentage on total viable cells of the MACS CD45+ selection, but differed significantly in their general occurrence between the two groups. Total RNA and DNA-concentration in the CEC population did not differ between the two groups. Established protocols for DNA- and RNA-isolation ensured enough material for downstream analysis.

**Table 1:** Overview of characteristics of the cohort including clinical parameters as well as CEC count.

	Young (n = 11)	Old (n = 11)	p-value
Age	25.2 (± 2.72)	73.5 (± 11.36)	< 0.0001
Sex			
f	81%	28%	
m	19%	72%	
BMI (kg/m^2^)	21.74 (± 1.6)	25.58 (± 2.5)	0.0005
Heart rate (bpm)	79 (± 9.4)	66.9 (± 8.3)	0.1807
Smoking (%)	9.1	45.5	
Diabetes (%)	0	54.5	
Hyperlipidaemia (%)	0	45.5	
Hypertension (%)	0	81.8	
CVD diagnosis (%)			
CAD	0	90.9	
Others	0	9.1	
LVEF (%)	62 (± 4.1))	55.3 (± 16.1)	0.2074
NT-pro BNP (ng/l)	^a^	1294.8 (± 1818.0)	
CRP (mg/l)	^a^	11.32 (± 17.9)	
LDL (mmol/l)	^a^	2.1 (± 0.9)	
Treatment no. (%)			
Antiplatelet drug	0	72.7	
ACE inhibitor	0	36.3	
β-Blocker	0	90.9	
Diuretic	0	36.3	
Statine	0	72.7	
Metformin	0	36.4	
SGLT2 inhibitor	9.1	27.3	
Other CVD relevant	0	63.6	
CEC per ml blood	64.8 (± 84.5)	409.6 (± 418.1)	0.0217
% viable CEC on total cells after CD45+ MACS selection	0.0029 (± 0.005)	0.0007 (± 0.001)	0.1807
DNA concentration (ng/µl)	2.2 (± 1.1)	2.1 (± 2.3)	0.9606
RNA concentration(ng/µl)	17.5 (± 6.7)	18.4 (± 8.2)	0.7812

^a^Parameters not quantified.

**Figure 6:**
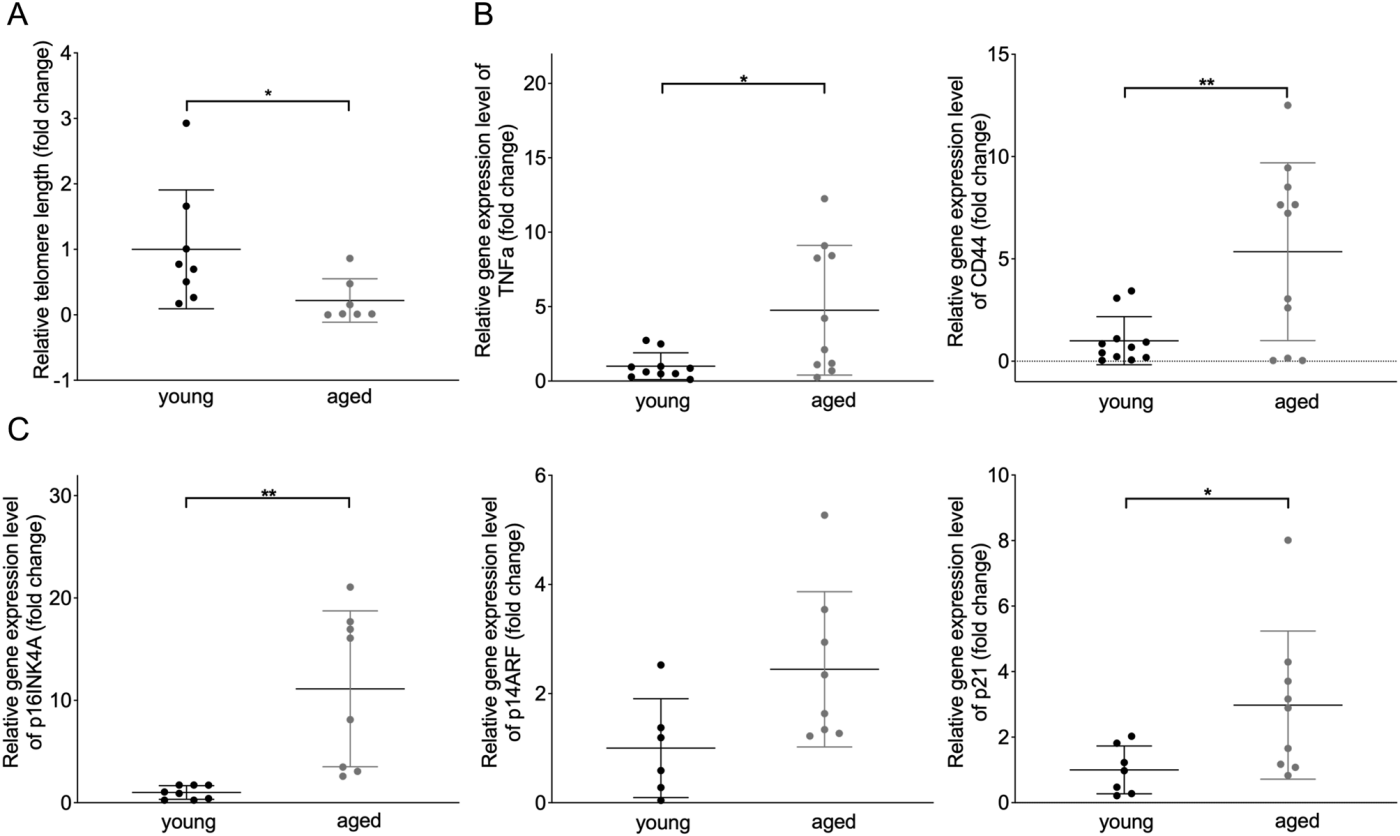
Quantification of senescence markers in circulating endothelial cells isolated from young individuals and aged patients with CVD. (**A**) Quantification of relative telomere length of young volunteers and aged individuals with CVD. n = 8 *P = 0.0386. (**B**–**F**) Relative gene expression levels of CD44, TNFα, p16INK4A, p14ARF and p21 between young and aged individuals. CD44 n = 11 **P = 0.0044, TNFα n = 10 *P = 0.0156, p16INK4A n = 8 **P = 0.0022, p14ARF n = 8 P = 0.0511 and p21 n = 9 *P = 0.0441.

Having established various expression-based EC senescence markers in vitro (Figs. 2, 3), we next assessed these markers in CECs. Unfortunately, staining’s and morphometric analyses were not possible on CECs due to difficulties with culturing ageing CECs from elderly volunteers. However, we could show that CECs from elderly people present with shortened telomeres, increased SASP-related gene expression signature (CD44 and TNFα) and increased expression of cell cycle regulator genes p16ink4a, p21, but not p14ARF (Fig. 6).

With these experiments we could show that CEC can be isolated from blood samples and that these cells have an increased expression of senescence associated genes in the aged patients compared to the young controls.

## Discussion and conclusion

The complexity of EC senescence physiology as well as difficulties with accessing material for functional studies hampers EC senescence research in various fields.

**Figure 7:**
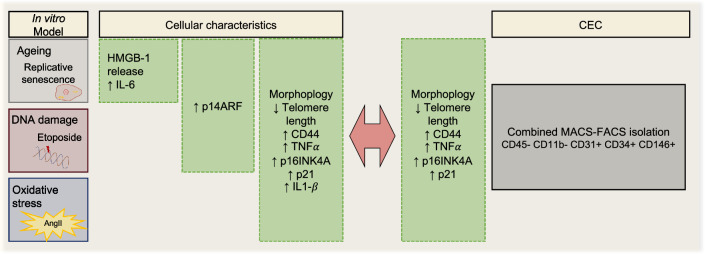
Summarizing observations.

Here, we provide first evidence of potential usability of circulating endothelial cells as reflections of vascular EC senescence, dysfunction and ageing. Employing a new combined MACS-and FACS-based CEC isolation method, we could isolate sufficient material to show increased EC senescence marker expression in elderly as compared to young individuals. The here observed results are displayed in Fig. 7, enabling to compare the observations from endothelial cell in vitro senescence to those characteristics observed in CEC.

The chosen in vitro models mimic different physiological stimuli that lead to cellular senescence: the replicative senescence reflects senescence observable during aging and is also known as cellular aging. The Etoposide model uses a chemotherapeutic that induces DNA-damage by interacting with topoisomerase II and thereby promoting DNA-double strand breaks^27^. In line with that DNA-damage effect, we observe high amount of γH2Ax foci in this model. In contrast, the Ang II model was used to mimic oxidative stress-induced senescence and serves a physiological stimulus as it is a part of the renin-angiotensin system playing a role in cardiovascular dynamics, especially in vasoconstriction^28,29^. All these models have been used before to study cellular senescence^30–33^. However, we here present the first comprehensive comparison of the three models with regard to various senescence markers and characteristics: Morphological changes, inhibition of proliferation and SA-β-gal-activity were observed for all models, as previously described in other cell types^19,30–32,34–37^. Also, the influence on the p53-p21 pathway was also already described for the models^31,36^. However, to our knowledge, we are the first to report release of HMGB-1 in replicative senescent cells but not in the other models. Upon senescence or cellular stress, HMGB-1 is shuffled into the extracellular space, where it binds amongst others cell surface RAGE and TLRs. This binding then initiates signaling, that results in expression of inflammatory cytokines including IL-6^22,23^. In line with that mechanism, we observe induction of IL-6 specifically in HMGB-1-releasing replicative senescent cells only. Release of HMGB-1 is mediated by the p53- pathway^38^. As this p53-pathway was fully upregulated in the etoposide model with an increased expression of p14ARF and p21, we suggest that HMGB-1 release is inhibited by other pathways in this setting. In turn, the lack of p14ARF regulation in the AngII model might explain the missing influence on HMGB-1 and IL-6.

In fact, AngII treatment likely induces EC senescence by a different pathway, namely activation of p21 through RAS rather than p14ARF^39^. AngII has previously been described to promote vascular senescence via an Angiotensin II receptor type 1 (AT1-receptor)-mediated inhibition of cyclin dependent kinase complexes and subsequent overexpression of p21^28^. In line with that, we observe increased p21 expression with unchanged p14ARF levels. However, the exact mechanism of p21 upregulation in our setting remains to be established. All these observations lead to the conclusion, that even though the models have many features in common, they also have major differences. This supports the need of choosing the right models to study senescence in a specific context.

The vascular endothelium in healthy humans is a dynamic barrier with a tight homeostasis of for example paracrine and autocrine regulators^1,40,41^. As soon as this tight homeostasis spins out of control, the endothelium becomes dysfunctional^42^. Often also observed in elderly, this endothelial dysfunction underlies clinical complications such as arterial stiffening or reduced endothelial-dependent dilation^42^. Recent histological studies show EC senescence in atherosclerotic plaques in individuals with ischemic heart disease, in the pathogenesis of heart failure, in aortic aneurysm, but also in aged healthy humans^43–45^. However, so far little is known about the exact role of EC senescence in CVD-related to the vasculature, except for that it is linked to a reduction in the regenerative and angiogenic capacity^1^.

Confirming earlier histological studies showing increased vascular EC senescence, we here observe enhanced expression of EC senescence markers in isolated circulating endothelial cells of elderly people.

Circulating cells in general are a new emerging field in the biomarker research, for example circulating tumor cells in certain cancer types^46^. In the vasculature, nonhematological circulating cells with endothelial identity can be detected, namely circulating endothelial cells^14^. These cells are released from the vasculature upon vascular damage^12^ and can be linked to multiple disorders as myocardial infarction or heart failure with preserved ejection fraction (HFpEF)^12,13^. So far, the direct cell count of CEC has been used as a biomarker for endothelial damage, but between different studies a wide range of CEC counts in (healthy) individuals can be observed^16^. This wide range might on the one hand be explained by the very low cell numbers obtained, their heterogeneous phenotype and the diversity in approaches used to isolate CECs^16^. Our data reflect previous studies indicating higher CEC cell counts in diseased individuals than in young healthy controls in regard to total cell count^12,13,16,47^, but in the context of living cells, the counted CEC did not differ between the groups. The conclusion if the higher total count is based on disease presence or progression, the dysfunctionality of the endothelial cells or the age is limited by the absence of an age-matched healthy cohort as well as by the missing quantification of endothelial function of the participants of the study. However, rather than assessing sole CEC quantity, CEC characteristics should be integrated in current biomarker development efforts and consistent protocols for CEC isolation need to be established. Here, we present a first attempt of CEC characterization with regard to senescence marker assessment. As CEC and EC senescence can both be found in aged-individuals or CVDs, CEC senescence markers may not only provide (adjuvant) biomarker potential, but may even prove as biologically relevant replicates of in vivo EC senescence, and present a valuable tool for functional studies and elucidate their role in the pathophysiology of CVDs.

In our study, we had access to aged volunteers with CVD co-morbidities and healthy young controls. The aged volunteers with CAD and HFpEF^48,49^, diseases associated with an endothelial dysfunction, presented with the expected CVD risk factors as smoking, hypertension or diabetes^50^. The established protocol to isolate CEC offers the great possibility to directly study this cell type in EDTA-blood samples. The combination of MACS-FACS-technology, described here, importantly enables a precise isolation. The CD45 negative pre-selection via MACS isolation removed among other cell types lymphocytes and leucocytes enabling the enrichment of CEC for FACS analysis. The selection used here also ensured to differentiate EPCs from CEC. Previous published protocols used a less stringent isolation^12,13,16,47,51–53^. The achieved RNA and DNA-quantity was sufficient to perform downstream analysis of some senescence-associated measurements. But, the inability of the cells to adhere as well as the low cell count prevented to perform all quantifications as in the in vitro models. Therefore, a conclusion whether senescence in CEC is replicative, related to DNA damage or oxidative stress is not possible as the obtained markers were commonly regulated in all three in vitro models and further investigations need to be conducted. Due to the low sample size (n = 11 aged volunteers and controls) and low material, we could not correct for co-morbidities in our analysis and cannot draw conclusions on whether EC senescence marker induction observed in elderly in this study is solely based on the age of the volunteers. A biased expression rather by CVD-comorbidities or even an interplay of both, age with CVD-comorbidities, is possible. To overcome this limitation, comparing with a group of elderly, healthy subjects would be meaningful, as it would allow differentiation between changes associated with aging and those associated with the disease. Further, an influence by either the risk factors or the CVD-treatment cannot be excluded as they are not present in the control group. In addition, it is known that some of treatments influence endothelial cell function. A therapy with Statin for example leads to increased levels of EPC^54^. Moreover, quantifying the endothelial dysfunction of the participants would allow conclusions to be drawn regarding whether the regulation of senescence-associated genes could also be related to the presence of endothelial dysfunction. While previous studies have shown a correlation between endothelial dysfunction and senescence, as well as between endothelial dysfunction and the occurrence of CEC, further connections have not yet been established^13,21^. Although the disease status and symptoms of the elderly, diseased patients with hypertension, diabetes, and CAD suggest the presence of endothelial dysfunction, this should be confirmed, for example, through measurement of the flow-mediated dilation in the forearm. This might further allow conclusions whether, CEC quantification and phenotyping as biomarker is more sensitive than the currently performed measurements. Yet, taken together our results provide first fundamental data for future research into CEC senescence to study not only their role in diseases, but also as a biomarker of in vivo EC function and vascular ageing or disease progression. Future studies should enable to focus on specific diseases. We further recommend to introduce an additional age-matched control group without CDV related co-morbidities to allow conclusions if the regulation is either age or disease related. The CEC isolation and genotyping might then present novel insights into vascular aging and the pathophysiology of diseases to use as biomarkers or develop possible treatment options.

In conclusion, we here provide first proof of CEC senescence detection in elderly as compared to young volunteers. This hints towards the exciting opportunity to exploit CEC senescence as potential reflection and biomarker of vascular endothelial function and ageing in vivo.

### Supplementary Information

Supplementary Information.

## Data Availability

The datasets used and analyzed during the current study are available from the corresponding author on reasonable request.

## Abbreviations

AngII: Angiotensin II
AT-1 receptor: Angiotensin II receptor type 1
BrdU: Bromodeoxyuridine
BSA: Bovine serum albumin
CAD: Coronary artery disease
CEC: Circulating endothelial cells
CVD: Cardiovascular disease
EC: Endothelial cell
EDTA: Ethylenediaminetetraacetic acid
EPC: Endothelial progenitor cells
FACS: Fluorescence activated cell sorting
γH2A.X: Phosphorylated histone H2A.X
HFpEF: Heart failure with preserved ejection fraction
HMGB-1: High mobility group box-1
HUVEC: Human umbilical vein endothelial cells
IL-1β: Interleukin-1 beta
IL-6: Interleukin-6
MACS: Magnetic beads associated cell sorting
NS: Non-senescent
PBS: Phosphate buffered saline
PDL: Passage doubling level
qRT-PCR: Quantitative real time polymerase chain reaction
RAGE: Receptor for advanced glycation endproducts
ROS: Reactive oxygen species
RS: Replicative senescent
SA-β gal: Senescence-associated beta galactosidase
SASP: Senescence-associated secretory phenotype
SGLT-2: Sodium/glucose cotransporter-2
TLR: Toll-like receptor
TNF-α: Tumor necrosis factor alpha
vWF: Von Willebrand factor
