# Supplementary material for: Isolation of circulating endothelial cells provides tool to determine endothelial cell senescence in blood samples
Source: Sci Rep. 2024 Feb 21;14:4271. doi: 10.1038/s41598-024-54455-5 (PMC10882010; doi:10.1038/s41598-024-54455-5)
Supplement: Supplementary file 1 — Supplementary Information. [file 41598_2024_54455_MOESM1_ESM.pdf]

Supplementary information

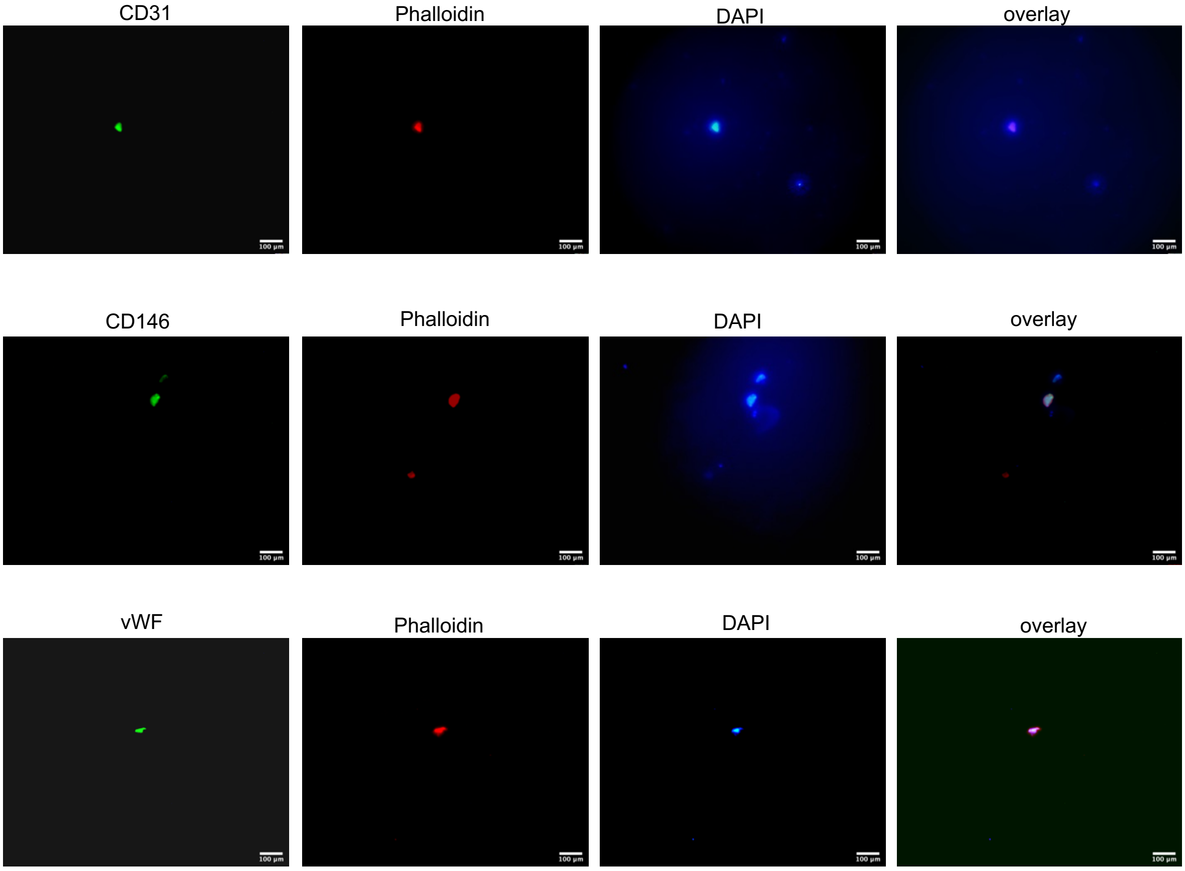

Supplemental Figure 1: Immunofluorescence staining of sorted circulating endothelial cells from young donors plated on fibronectin and stained for either CD31, CD146 or vWF. *Cells can be stained positive for CD31, CD146 and vWF (green). Co-staining's are performed for Phalloidin (red) and DAPI (blue) to visualize cell area and cell nuclei.*

Table S 1 Primer Sequences

Gene		Sequence
p16INK4A	for	GGGGGCACCAGAGGCAGT
	rev	GGTTGTGGCGGGGGCAGTT
p14ARF	for	CCCTCGTGCTGATGCTACTG
	rev	CATCATGACCTGGTCTTCTAGGAA
CD44	for	TGGCACCCGCTATGTCGAG
	rev	GTAGCAGGGATTCTGTCTG
TNFα	for	GGCGTGGAGCTGAGAGATA
	rev	CAGCCTTGGCCCTTGAAGA
IL1β	for	AAAGCTTGGTGATGTCTGGTC
	rev	GGACATGGAGAACACCACTTG
IL-6	for	GGCACTGGCAGAAAACAACC
	rev	GCAAGTCTCCTCATTGAATCC
GAPDH	for	TGCACCACCAACTGCTTAGC
	rev	GGCATGGACTGTGGTCATGAG
FBX07	for	GCTCGCACCTGAGGCAGTCC
	rev	GTCTCTTCATCTCCAGTGAGGGG

Table S 2 Antibodies used for Immunofluorescence staining

Antibody	Company	Order number	Dilution
Phalloidin-TRITC	Sigma	P1951	1:100
CD31	Cell Signaling	3528S	1:100
HMGB-1	Santa Cruz	sc-135809	1:1000
γH2Ax	Cell Signaling	9718	1:200
CD146	abcam	75769	1:250
vWF	abcam	6994	1:400

Table S 3 Antibodies used for FACS

Antibody	Company	Order number	Dilution
CD45	Miltenyi Biotec	P1951	1:50
CD11b	Miltenyi Biotec	3528S	1:50
CD31	Miltenyi Biotec	sc-135809	1:50
CD146	Miltenyi Biotec	10883-1-AP	1:50
CD34	Miltenyi Biotec	9718	1:50
